# Nitrification kinetics, N_2_O emission, and energy use in intermittently aerated hybrid reactor under different organic loading rates

**DOI:** 10.1007/s13762-022-04715-6

**Published:** 2022-12-20

**Authors:** O. Zajac, M. Zubrowska-Sudol

**Affiliations:** Department of Water Supply and Wastewater Treatment, Faculty of Building Services, Hydro and Environmental Engineering, Warsaw University of Technology, Nowowiejska 20, 00-653 Warsaw, Poland

**Keywords:** Ammonia oxidation rate, Intermittent aeration, Nitrite oxidation rate, Energy, Nitrous oxide, Biofilm, Activated sludge

## Abstract

This study investigated the impact of intermittent aeration strategies and reduction in the reactor’s organic and nitrogen loading rates on the course of particular stages of the nitrification process, as well as energy consumption and N_2_O emissions in a hybrid reactor with nitrification/denitrification. Each of the analysed series revealed the greatest ammonia oxidation activity in activated sludge flocs. The highest activity of nitrite nitrogen oxidation was demonstrated in the case of biofilm. A reduction in the reactor’s organic and nitrogen loading rate value had a greater effect on changes in the activity of ammonia-oxidizing bacteria than nitrite-oxidizing bacteria. In a system where the operation of air pumps was controlled through switching them and off according to the adopted ratio between non-aerated and aerated sub-phase times and the assumed oxygen concentration, a reduction in the duration of aerated sub-phases caused no decrease in energy use for aeration. Lower N_2_O emission was recorded when the reactor operated with a longer duration of aerated sub-phases.

**Supplementary Information:**

The online version contains supplementary material available at 10.1007/s13762-022-04715-6.

## Introduction

The twenty-first century has brought dynamic development in the scope of biological nutrient removal (BNR). It is manifested in a number of technological solutions applied in wastewater treatment plants (WWTP). Next to the widely known activated sludge technology, biofilm systems are already in common use. Hybrid technology (Integrated Fixed-Film Activated Sludge—IFAS) is also becoming increasingly popular. This technological solution employs combined activated sludge and biofilm developing on moving or fixed beds with a large active surface. It considerably increases the amount of biomass in the hybrid reactor in comparison with reactors operating only based on activated sludge. Biofilm can also provide for longer biomass retention time (SRT). This contributes to the development of slow-growing nitrifiers, therefore improving the efficiency of the nitrification process (Shao et al. 2017). In comparison with conventional wastewater treatment systems, IFAS systems guarantee a number of advantages. They include: high organics and nitrogen removal efficiencies, low sludge production rates, and lack of problems with sludge bulking. They have one considerable drawback, however, namely higher energy use in comparison with other systems (Singh et al. 2017). According to the cited authors, solving this problem requires the application of the intermittent aeration (IA) strategy that decreases unitary energy use with no substantial effect on nutrient removal efficiency. One of the important aspects of IA is a compromise between energy reduction and the achieved nutrient removal efficiency.

The literature regarding hybrid systems with nitrification/denitrification (N/D) applying the IA strategy is still scarce. Next to Singh et al. (2017) cited above, the issue was also investigated by Bhatia et al. (2017) and Singh et al. (2019) (Table 1). Bhatia et al. (2017) studied the effect of IA on the suppression of nitrite-oxidizing bacteria (NOB), and Singh et al. (2019) analysed the efficiency of IFAS operating with three different IA variants with respect to pre-set high aeration rate.

**Table 1:** Comparison of different hybrid reactors with intermittent aeration

Type of reactor	Operation conditions			Removal efficiences				Refs.
	DO [mg O_2_/L]	Intermittent aeration (Oxic/Anoxic) [min]	R	COD [%]	N–NH_4_^+^ [%]	TN [%]	TP [%]	
IFAS reactor operating in conventional ASP configuration (aeration tank followed by settling tank) Biofilm carriers: Biotextil Cleartec® media Activated sludge concentration: 2 ± 0.2 gMLSS/L	~ 4.5^a^	150/30 120/60 90/60	1/5 1/2 2/3	92.71 89.87 86.96	100 100 94.8	58 63 55	81.4^x^ 66.1^x^ 66.4^x^	Singh et al. (2019)
IFAS reactor operating in conventional ASP configuration (aeration tank followed by settling tank) Biofilm carriers: Biotextil Cleartec® media Activated sludge concentration: 1.28–1.45 gMLSS/L	∼ 3.0^b^	150/30 120/60 90/60	1/5 1/2 2/3	96 97 96	97.2 85.8 80.3	77.8 73.5 74.1	80.4 63.3 43.6	Singh et al. (2017)
IFAS-MBSBBR Biofilm carriers: EvU-Pearl® Activated sludge concentration: 1.71 ± 0.23 gMLSS/L	1.5^c^	30/10 20/10 20/10 20/10	1/3 1/2 1/2 1/2	96.55 ± 0.95* 96.32 ± 0.70* 96.21 ± 0.16* 96.85 ± 0.85*	89.80 ± 4.34*,** 88.21 ± 5.58*,** 89.02 ± 3.14*,** 89.98 ± 2.13*,**	79.27 ± 2.77* 79.97 ± 2.12* 79.69 ± 2.61* 79.05 ± 2.00*	89.17 ± 1.59* 89.12 ± 2.86* 89.48 ± 5.90* 89.41 ± 2.09*	This study

^a^Air pumps in aerated phases operate with a constant air flow of 110 m^3^/h; ^b^Air pumps in aerated phases operate with a constant air flow of 75 m^3^/h; ^c^The operation of air pumps in aerated phases is controlled by the pre-set level of oxygen concentration (the air pumps switch off when the level exceeds 1.5 mg O_2_/L, and switch on when it falls below 1.5 mgO_2_/L); ^x^ with chemical precipitation; * values calculated in accordance with the methodology provided by Podedworna et al. (2012); ** the determination of the efficiency of N–NH_4_^+^ removal considered the N–NH_4_^+^ load supplied to the reactor with raw wastewater, and N–NH_4_^+^ load developing in the course of treatment as a result of the process of ammonification of organic nitrogen

The cited papers therefore primarily focused on the analysis of the effect of IA on the efficiency of operation of IFAS. This study also presents an attempt to determine the effect of a decrease in the reactor’s organic (L_COD_) and nitrogen (L_N_) loading rate on the efficiency of removal of pollutants.

As already mentioned before, IA applied in IFAS systems is an alternative that permits a decrease in the operation costs related to energy used for aeration, with simultaneous maintenance of high efficiency of nutrients removal. Focusing on this issue, this study also involved an analysis of the amount of energy used for aeration in different variants of IA. The analysis pointed to a strategy allowing for a decrease in the operation costs of IFAS with simultaneous maintenance of high efficiency of nutrients removal. It is also worth mentioning that this energy is largely used in the nitrification process. An important element of the study is therefore the analysis of the effect of the aforementioned factors on the ammonia oxidation rate (AOR) and nitrite oxidation rate (NitOR) for activated sludge and biofilm, and in the case of a hybrid combination of these two forms of biomass. The experiment was conducted from May 2019 to August 2020 at the Warsaw University of Technology (Warsaw, Poland).

In selected series of the experiment, the effect of IA on the emission of nitrous oxide (N_2_O) was additionally determined. It is a strong greenhouse gas that primarily originates as a by-product of ammonia oxidation. Due to its high stability, the gas can persist in the atmosphere for even more than 120 years (Schreiber et al. 2012), causing irreversible changes in the environment. Over the recent years, Integrated Fixed-Film Activated Sludge–Moving-Bed Sequencing Batch Biofilm Reactors (IFAS-MBSBBR) have become increasingly popular due to their high treatment efficiency combined with low operation costs and simplicity of operation (Sabba et al. 2018). It is therefore important to investigate their effect on the environment, and the issue of climate warming Earth is facing as a result of human activity.

## Materials and methods

### Reactor set-up and operation

**Fig. 1:**
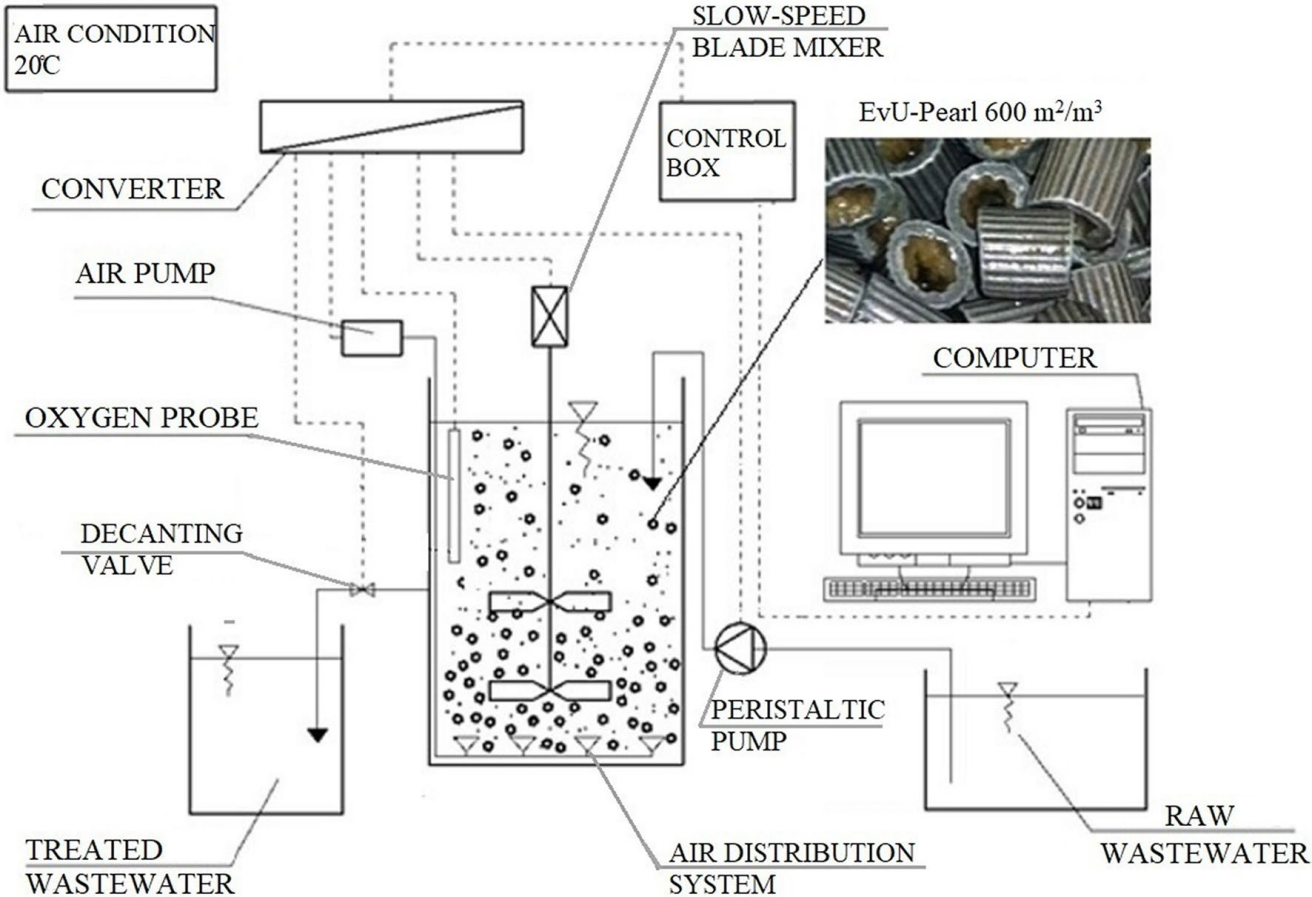
Schematic of the reactor with accessories

The study was conducted on a laboratory model of IFAS-MBSBBR with an active volume of 28L (Fig. 1). The system was equipped with the following: a slow-speed overhead stirrer R-50D by CAT, air pumps, and an air distributing system placed at the bottom of the reactor, peristaltic pump Ismatec Ecoline, optical oxygen probe Oxymax COS61D connected to a transmitter Liguiline CM442 (measurement of dissolved oxygen concentration and temperature), and an automatic control system (DreamSpark Premium software based on the SCADA system).

The study was launched on an operating IFAS-MBSBBR for nitrogen, carbon, and phosphorus removal with biomass developed in the form of biofilm on moving carriers EvU-Pearl (0.43 gSS/m^2^) and activated sludge (1.758 gMLSS/L). The carriers had a specific surface area of 600 m^2^/m^3^ and constituted 25% of the active volume of the reactor, i.e. 7 L.

Throughout the experiment, the average concentration of activated sludge was maintained at the level obtained at the beginning of the experiment, i.e. approximately 1.7 gMLSS/L.

IFAS-MBSBBR operated in three 8-h cycles per day, with the following subsequent phases: 1st phase without aeration—50 min, 1st phase with aeration (with intermittent aeration)—190 min, 2nd phase without aeration—30 min, 2nd phase with aeration (with intermittent aeration)—150 min, sedimentation—50 min, decantation—10 min. At the beginning of each of the phases without aeration, the reactor was fed synthetic wastewater, with a composition simulating municipal wastewater. It was prepared based on dechlorinated tap water and a mixture of the following: ammonium acetate 225 mg/L; peptone 135 mg/L; starch 45 mg/L; glucose 45 mg/L; glycerine 0.049 ml/L; NaHCO_3_ 125 mg/L; Na_2_HPO_4_ 15 mg/L; KH_2_PO_4_ 4.5 mg/L with a volume of 10L per cycle (S. I., II., IV.) and 6.6L per cycle (S.III.). The composition of raw wastewater was the same in all series; COD: 511.61 ± 7.86 mgO_2_/L, TN: 60.81 ± 1.74 mgN/L, TKN: 59.33 ± 1.56 mgN/L, N–NH_4_^+^: 39.04 ± 1.30 mgN–NH_4_^+^/L; N–NO_3_^−^: 1.47 ± 0.58 mgN–NO_3_^−^/L, P-PO_4_^3−^: 7.61 ± 0.26 mgP–PO_4_^3−^/L, pH: 7.6–7.8. A new portion of wastewater was prepared: i) daily, with the exception of weekends (S.I., II., IV.); ii) in accordance with the methodology described in point 2.2. (S.III.).

In all series, dissolved oxygen (DO) concentration in phases with aeration was maintained at 1.5 mgO_2_/L. The temperature in the reactor was kept at a constant level of 20 °C by means of external air-conditioning.

### Study series

Four series were designated in the experiment (I–IV), differing in the applied intermittent aeration strategy and reactor’s organic loading rate (L_COD_) and nitrogen loading rate (L_N_) (Table 2).

**Table 2:** Reactor operation series

Series	Duration [days]	Organic Loading Rate [gCOD/m^3^·d] (L_COD_) Nitrogen Loading Rate [gN/m^3^·d] (L_N_)	Description of aeration strategy		
			Times of aerated t_1_ and non-aerated t_2_ sub-phases [min]	Ratio between times of non-aerated and aerated sub-phases (R)	O_2_ concentration during aerated sub-phases [mg/L] (DO)
I	0- 131 (22–44)*	L_COD_ = 536 L_N_ = 64	t_1_ = 30 min, t_2_ = 10 min	1/3	1.5
II	132–375 (154–226)*	L_COD_ = 536 L_N_ = 64	t_1_ = 20 min, t_2_ = 10 min	1/2	
III	376–443	L_COD_ = 402 L_N_ = 48	t_1_ = 20 min, t_2_ = 10 min	1/2	
IV	444–517	L_COD_ = 536 L_N_ = 64	t_1_ = 20 min, t_2_ = 10 min	1/2	

*Failure period caused by an uncontrolled loss of activated sludge as a result of damaged electromagnetic valve

The study was launched on an operating IFAS-MBSBBR ensuring highly efficient nitrification (96.97%, N–NH_4_ concentration in treated wastewater 0.29 mg/L). The efficiency of removal of organic compounds and nitrogen and phosphorus compounds reached 97.23%, 80.78%, and 71.25%, respectively (COD, TN, and P concentration in treated wastewater 14.20 mgO_2_/L, 12.30 mgN/L, 2.55 mgP/L).

Between series I (S.I.) and II (S.II.), the duration of aerated sub-phases was reduced from 30 min to 20 min. The L_COD_ and L_N_ values were at the same level at the time.

Due to the outbreak of the Covid-19 pandemic, changes have been introduced in the methodology of operation of IFAS-MBSBBR. They aimed at the maintenance of stable operation of the system for as long as possible. Due to the further development of the pandemic and restrictions introduced by the government, a decision was taken to reduce the reactor’s organic and nitrogen loading rate through a decrease in the volume of wastewater supplied to the reactor from 10 L to 6.6 L per cycle. This way, a new portion of raw wastewater could be prepared only once in 5 days. In reference to the introduced changes, series III (S.III.) was designated.

On cancelling part of restrictions, the reactor’s organic and nitrogen loading rate returned to that assumed at the beginning of the experiment—series IV (S.IV.).

During series I, II, and IV twice a week, and in series III once a week, raw and treated wastewater was analysed in the following scope: COD, TN, N–NO_2_^−^, N–NO_3_^−^, P–PO_4_^3−^, alkalinity, pH, and determination of the activated sludge concentration. N–NH_4_^+^ was controlled daily. The exception was series III where the analyses were conducted once a week.

### Batch tests of the course of ammonia and nitrite oxidation

The determination of the effects of the operation conditions of IFAS-MBSBBR on the ammonia and nitrite oxidation process conducted by microorganisms inhabiting activated sludge flocs and those developing in the form of biofilm employed batch tests: Ammonia Utilization Rate Test (AUR) and Nitrite Utilization Rate Test (NitUR). The tests were conducted in two different variants:

1. V.I.—for a strictly specified level of oxygen concentration—1.5 mgO_2_/L (V.I.1.5)—and 1.0 mgO_2_/L (V.I.1.0)—the selection of the former corresponded with DO concentration maintained throughout the experiment in the aerated phases of the cycle in IFAS-MBSBBR. The selection of the latter was preceded by literature review according to which low DO concentrations may cause NOB bacteria suppression and contribute to favouring bacteria showing greater activity in such conditions (Park et al. 2002).
  The tests were performed for each of the forms of biomass sampled from IFAS-MBSBBR separately, namely activated sludge (SB) and biofilm (B), and for their combination—hybrid (H).
2. V.II.—with the assumption that oxygen concentration will be approximate to saturation in the conditions of 20 °C—in this case it was important that DO concentration does not constitute a factor limiting the ammonia or nitrite oxidation rate (at too low DO, such a phenomenon occurs in biofilm) (Pal et al. 2012).

In variant II, the tests were conducted for activated sludge (SB) and biofilm (B).

Methodological assumptions shared by both the variants were as follows:

(A) Initial N–NH_4_^+^ concentration in the AUR_V.I._ and AUR_V.II._ test equal to 15 mgN–NH_4_^+^/L and N–NO_2_^−^ concentration in NitUR_V.I._ and NitUR_V.II._ equal to 15 mgN–NO_2_^−^/L.
(B) Temperature – 20 °C.
(C) In tests for the designated forms of biomass: activated sludge concentration at a level of approximately 0.9 gMLSS/L, percent moving bed content at 25% of the active volume of the reactor.

It was assumed that batch tests will be conducted when comparable efficiency of the nitrification process is recorded for at least 10 subsequent days after changes in particular series in IFAS-MBSBBR.

### N_2_O emission

During series I and II, measurements of nitrous oxide emission were conducted using a N_2_O-R Microsensor (Unisense, Denmark). The analysis covered measurements selected from 3 days of stable operation of the IFAS-MBSBBR. It was assumed that the N_2_O emission coefficient (${\text{E}}_{{\text{N}_\text{2} \text{O}}}$) would be calculated as mass of nitrous oxide (${\text{M}}_{{\text{N}_\text{2} \text{O}}}$) emitted in the reactor operation cycle divided by nitrogen loading rate (L_N_), modifying the methodology proposed by Al-Hazmi et al. (2020) (Eq. 1).

1 $$E_{{{\text{N}}_{2} {\text{O}}}} = \frac{{M_{{{\text{N}}_{2} {\text{O}}}} }}{{L_{N} }} \times 100\% \left[ \% \right]$$

${\text{M}}_{{\text{N}_\text{2} \text{O}}}$ [mg N–N_2_O/L·d] was calculated by integrating the rate of N_2_O emission (${\text{r}}_{{\text{N}_\text{2} \text{O}}}$ [mg N–N_2_O/L·d]) over the unit time (dt[d]) and dividing by total reaction time (t[d]) (Eq. 2).

2 $$M_{{{\text{N}}_{2} {\text{O}}}} = \int {\frac{{r_{{N_{2} O}} \times dt}}{t}\left[ {mg\;{\text{N}} - {\text{N}}_{2} {\text{O}}/L \cdot d} \right]}$$

### Analytical methods

Concentrations of COD, TN, N–NH_4_^+^, N–NO_2_^−^, N–NO_3_^−^, P-PO_4_^3−^ were analysed spectrometrically according to APHA Standard Methods (APHA 2005) using cuvette tests (Hach Lange GmbH) and a DR 3900 spectrophotometer (Hach Lange GmbH, Berlin, Germany). The determination of mixed liquor suspended solids (MLSS) and mixed liquor volatile suspended solids (MLVSS) employed gravimetric methods in accordance with Polish standard PN-EN 872:2007. Measurement of total volatile solids in biofilm was performed in accordance with the Polish standard by calculating weight loss. The biofilm was mechanically removed from the carries.

Free ammonia (FA) and free nitrous acid (FNA) were calculated according to Eqs. (3) and (4) (Anthonisen et al. 1976)

3 $${\text{FA}} = \frac{17}{{14}}{ } \times { }\frac{{{\text{total }}\;{\text{ammonia}}\;{\text{ as}}\;{\text{ N }}\left( {\frac{{{\text{mg}}}}{{\text{L}}}} \right) \times 10^{{{\text{pH}}}} }}{{{\text{e}}^{{\left( {6344/273 + {\text{T}}^\circ {\text{C}}} \right)}} + 10^{{{\text{pH}}}} }}{ }\left[ {{\text{mgFA}}/{\text{L}}} \right]$$

4 $${\text{FNA}} = \frac{46}{{14}}{ } \times { }\frac{{{\text{N}} - {\text{NO}}_{2}^{ - } \left( {\frac{{{\text{mg}}}}{{\text{L}}}} \right)}}{{{\text{e}}^{{\left( { - 2300/273 + {\text{T}}^\circ {\text{C}}} \right)}} \times 10^{{{\text{pH}}}} }}{ }\left[ {{\text{mgFNA}}/{\text{L}}} \right]$$

### Statistical analysis

The statistical analysis of the obtained results regarding treated wastewater quality and efficiency of removal of particular pollutants was conducted in program Statistica 13.3PL. A RiR Tukey test was applied for the determination of the significance of differences between the analysed variables (*p*-value smaller than 0.05 indicated a statistically significant difference).

## Results and discussion

### Organics, nitrogen, and phosphorus removal

According to the data presented in Table 3, irrespective of the duration of aerated sub-phases and values of L_COD_ and L_N_, throughout all series a comparable high efficiency of the nitrification process was obtained, namely E_N_ = 89.31 ± 4.34% (*p* > 0.620). N–NH_4_^+^ concentrations in wastewater discharged from IFAS-MBSBBR were approximate to 0.20–1.75 mgN–NH_4_^+^/L. The statistical analysis showed a significant differences between N–NH_4_^+^ values for S.I.–S.II. (*p* = 0.000008) and S.I.–S.III. (*p* = 0.005949).

**Table 3:** Effluent characteristics of IFAS-MBSBBR

Parameter			Series			
			I	II	III	IV
COD*	mgO_2_/L	Min	11.50	12.43	18.00	10.60
		Max	28.60	26.00	20.00	24.40
		Average	17.63 ± 4.82	18.67 ± 3.49	19.36 ± 0.78	16.27 ± 4.40
TN^*^	mgN/L	Min	10.90	10.50	10.30	10.00
		Max	15.87	14.70	13.70	14.50
		Average	12.70 ± 1.64	12.29 ± 1.36	12.02 ± 1.41	12.25 ± 1.25
TKN	mgN/L	Min	1.26	1.09	5.15	3.98
		Max	10.92	11.51	8.94	7.97
		Average	6.04 ± 2.52	7.06 ± 3.35	3.01 ± 1.08	2.85 ± 1.36
N–NH_4_^+ *^	mgN–NH_4_^+^/L	Min	0.20	0.32	0.29	0.20
		Max	1.75	1.75	1.59	1.55
		Average	0.55 ± 0.35	0.82 ± 0.32	0.83 ± 0.41	0.69 ± 0.30
N–NO_2_^− *^	mgN–NO_2_^−^/L	Min	0.01	0.02	0.00	0.01
		Max	0.87	0.20	0.26	0.44
		Average	0.08 ± 0.18	0.07 ± 0.06	0.09 ± 0.12	0.05 ± 0.12
N–NO_3_^− *^	mgN–NO_3_^−^/L	Min	3.17	2.87	4.50	4.13
		Max	12.40	9.88	7.10	9.07
		Average	6.59 ± 2.32	5.16 ± 2.35	5.48 ± 1.12	6.69 ± 1.49
TP *	mgP-PO_4_^3−^/L	Min	0.60	0.89	0.76	0.60
		Max	1.05	1.05	0.99	1.05
		Average	0.82 ± 0.11	0.87 ± 0.07	0.86 ± 0.10	0.80 ± 0.14
L_COD_		gCOD/m^3^·d	548.10 ± 10.45	545.24 ± 8.36	410.81 ± 0.44	543.43 ± 39.71
L_N_		gN/m^3^·d	65.69 ± 2.24	65.73 ± 0.85	47.97 ± 1.08	63.71 ± 1.24

*Courses of changes in the analysed indicators throughout the experiment are provided in Supplementary Information: Figs. 1–5

A highly efficient process of COD removal (E_COD_ = 96.52 ± 0.83%) was also achieved. The value of COD in treated wastewater did not exceed 29 mgO_2_/L in any of the series and remained far below the discharge standard in Poland (125 mgO_2_/L) (Regulation of the Minister of Marine Economy and Inland Navigation, 2019). The statistical analysis showed lack of significant differences between the values of E_COD_ and COD in the effluent for all of the series, pointing to the lack of effect of the analysed aeration strategies and L_COD_ on the efficiency of organics removal. In the conducted research series, no statistically significant changes were recorded in the efficiency of nitrogen and phosphorus compounds removal or in TN and TP concentrations in treated wastewater.

A comparison of results obtained in this study with research presented in Table 1 showed that at an identical value of the R indicator, the nitrification efficiency is approximately 11% lower than that determined by Singh et al. (2019). DO concentration and duration of particular aerated sub-phases in the cited paper were, respectively, 3 times and 6 times higher than in this study, potentially affecting the analysed process.

The efficiency of TN removal was higher than in research conducted by Singh et al. (2019), although TN concentration in the effluent was at an approximate or somewhat higher level in comparison with the remaining papers cited in Table 1.

To sum up, IFAS-MBSBBR was characterized by high stability of contaminant removal irrespective of changes in the values of R, L_COD,_ and L_N_.

### Batch test results

The analysis of the quality of raw and treated wastewater showed a comparable efficiency of the nitrification process, irrespective of the duration of aerated sub-phases and values of L_COD_ and L_N_. In order to understand the role of microorganisms developed in the form of activated sludge flocs and those in the form of biofilm in particular stages of nitrification, batch tests were conducted, aimed at the assessment of the ammonia oxidation and nitrite oxidation rate. The tests were conducted for biofilm (B), activated sludge (SB), and combination of both these forms—hybrid (H). Their results are collected in Table 4 (detailed results of particular batch tests are presented in the Supplementary Information: Figs. 6–13). They provided the basis for the identification of the form of biomass showing the highest ammonia or nitrite oxidation activity, and determination of how the reactor operation conditions during particular series of the experiment contributed to changes in such values.

**Table 4:** Summary of batch test results

Parameter	DO [mg O_2_/L]	Series I			Series II			Series III			Series IV		
		SB	B	H	SB	B	H	SB	B	H	SB	B	H
Ammonia oxidation rate (AOR) mgN–NH_4_^+^/gVSS·h	nl	6.841	3.374	–	3.856	6.993	–	5.112	4.700	–	8.061	3.623	–
	1.5	3.806	1.478	2.614	4.555	1.733	3.102	–	–	–	2.887	1.504	1.911
	1.0	3.855	0.890	–	–	–	–	–	–	–	3.607	1.865	–
Nitrite oxidation rate (NitOR) mgN–NO_2_^−^/gVSS·h	nl	4.211	9.239	–	3.503	21.446	–	3.851	19.276	–	4.868	18.588	–
	1.5	2.275	4.786	2.577	2.516	7.024	5.249	–	–	–	1.960	6.778	3.641
NitOR/AOR mgN–NO_2_^−^/ mgN–NH_4_^+^	nl	0.62	2.76	–	0.91	3.07	–	0.75	4.10	–	0.60	5.14	–
	1.5	0.60	3.24	0.99	0.55	4.05	1.69	–	–	–	0.68	4.51	1.91

### Ammonia oxidation rate and nitrite oxidation rate depending on the form of biomass in the reactor

The objective of batch tests conducted at 1.5 mg O_2_/L (AUR_V.I.1.5._, NitUR_V.I.1.5._) was to compare the activity of particular groups of nitrification microorganisms developing in the form of activated sludge and biofilm, and in the case of both these biomass forms cooperating as a hybrid. A separate paper describing the composition of microorganisms inhabiting the biomass developing in the discussed reactor showed that the community of biofilm microorganisms was richer and more diverse than in the activated sludge (Godzieba et al. 2022).

In each of the analysed series, the highest AOR values were recorded for activated sludge. In comparison with values obtained for biofilm, it was higher: 2.58 times (S.I.), 2.63 times (S.II.), and 1.92 times (S.IV.), respectively, pointing to greater ammonia oxidation activity in activated sludge flocs than in biofilm. Microbiological analysis showed that in S.I. and S.II. *Rhodobacter*, considered a facultative anaerobic bacterium able to perform heterotrophic nitrification and aerobic denitrification, was present in the activated sludge with much higher relative abundance (Godzieba et al. 2022).

In research presented in the literature analysing the ammonia oxidation rate in hybrid systems, a higher value of the analysed indicator was obtained in tests conducted for moving carriers (Table 5) (Christensson et al. 2004; Onnis-Hayden et al. 2011; Regmi et al. 2011; Di Trapani et al. 2013). Onnis et al. (2011) and Regmi et al. (2011) observed more than 75% higher values of nitrification activity in tests conducted for biofilm in comparison with those observed for activated sludge. Another group of researchers evidenced that 85% of the nitrification activity took place on a suspended carrier (Christensson et al. 2004). Lack of similar dependencies in this study, however, may be related to the type of the analysed wastewater treatment system and DO concentration lower than in the cited papers (1.5 mgO_2_/L vs. 3–5 mgO_2_/L). It is also important that in the case of biofilm, oxygen diffusion plays a considerably greater role than in activated sludge. According to Shao et al. (2017), due to the diffusion resistance through biofilm, the oxygen half-saturation coefficient (K_O_) was up to 10 times higher in the biofilm than in the sludge (the higher the K_O_ value, the smaller oxygen transport in biofilm), leading to a considerable decrease in DO concentration of the already shallow depth of biofilm. In the case of NitUR tests conducted at 1.5 mgO_2_/L, the highest values of the analysed indicator in all series were recorded for biofilm. In reference to values determined for SB, they were 2.10 times (S.I.), 2.79 times (S.II.), and 3.46 times higher (S.IV.), pointing to higher nitrite oxidation activity in biofilm. A higher nitrite oxidation rate for biofilm in comparison with activated sludge was also recorded in the literature (Onnis-Hayden et al. 2011; Regmi et al. 2011; Shao et al. 2017). Regmi et al. (2011) showed NitOR for biofilm as much as 9.21 times greater than that for activated sludge. In microbiological analyses, in the case of biofilm, Onnis et al. (2011) and Shao et al. (2017) recorded higher NOB abundance. The microbiological analysis of biomass samples from this study is consistent with what the cited authors presented. The microbiological tests showed that *Nitrospiriota* and genus *Nitrospira* were much more numerous in the biofilm than in the activated sludge. This could have undoubtedly influenced the obtained NitOR values and suggested that biofilm was a better environment to develop nitrifiers (Godzieba et al. 2022).

**Table 5:** N–NH_4_^+^ and N–NO_2_^−^oxidation rate for different hybrid reactors

Aeration strategy	Unit	N–NH_4_^+^ oxidation rate			N–NO_2_^−^ oxidation rate			References
		AS	B	H	AS	B	H	
Continuous	mgNH_4_–N/gTS/h	2.64	5.89	4.77	–	–	–	Onnis-Hayden et al. (2011)
	mgNO_*x*_–N/gTS/h	2.02	5.87	5.16	–	–	–	
Continuous	mgNO_*x*_/gMLSS/h	1.72	4.97	–	0.82	7.55		Regmi et al. (2011)
Continuous	mgNH_4_^+^–N/gVSS/h	–	–	5.28 (COD:N = 10:1) 7.99 (COD:N = 5:1) 18.25 (COD:N = 3:1)	–	––	–	Shao et al., (2017)
		–	–		–	–	––	
		–	–		–	–	–	
Continuous	gNH_4_–N/m^3^/h	–	–	18.8–23	–	–	–	Christensson et al. 2004
	gNH_4_–N /m^2^/d	–	0.86–1.18	–	–	–	–	
Continuous	gNH_4_–N/m^2^·d	– – –	0.92 0.71 0.92	– – –	– – –	– – –	– – –	Di Trapani et al. 2013
	gNH_4_–N/gVSS h	2.05 1.27 2.57	– – –	– – ––	– – ––	– –– –	– – –	

In accordance with data presented in the literature (Onnis-Hayden et al. 2011; Regmi et al. 2011), it was expected that in tests conducted for the combination of both forms of biomass, the AOR and NitOR values would be higher, or at least approximate to the highest values recorded for SB or B. Nonetheless, in the case of AUR-H_V.I.1.5._ tests, in each series, AOR were approximately 1.46 (S.I.), 1.47 (S.II.), and 1.51 times (S.IV.) lower than the values observed for SB and were 1.77 (S.I.), 1.78 (S.II.), and 1.27 times (S.IV.) higher than values recorded in AUR-B_V.I.1.5_ tests. In reference to NitUR-H_V.I.1.5._ tests, in each series, the NitOR was 1.86 (S.I.), 1.34 (S.II.), and 1.86 times (S.IV.) lower than that determined for biofilm. These observations may point to competition for substrate and oxygen of ammonia-oxidizing bacteria (AUR tests) and nitrite-oxidizing bacteria (NitUR tests) in particular forms of biomass.

It is also worth emphasizing that although the reactor operation conditions during series II and IV were identical, the obtained values of the rate of the analysed processes were different. AOR for SB and H recorded in series IV decreased 0.63 and 0.62 times, respectively, in reference to the values observed in series II, and the value recorded for B constituted 86% of that from series II. In reference to the NitOR, the greatest difference between the values of the analysed indicator in series II and IV was evidenced for H. The recorded value was 1.44 times lower than that presented in series II. Also in the case of tests conducted in series IV for SB, the NitOR_V.I.1.5._ value was 1.28 times lower than in S.II. A temporary reduction in the reactor’s organic loading rate in series III caused changes in the ammonia and nitrite oxidation rate. Microbiological analysis also showed discrepancies in relative abundances, both in phyla and genera, while in both cases they were greater in the case of activated sludge (Godzieba et al. 2022). For example, *Rhodobacter* was present in the sample in series II, while in series IV it was already gone, which can contribute to differences in AOR values for SB and H.

### Effect of DO concentration on the activity of nitrification microorganisms

In series I and IV, the highest AOR by microorganisms inhabiting activated sludge flocs was observed in the case of unlimited oxygen concentration. Values recorded in tests at oxygen concentration of 1.5 mgO_2_/L and 1.0 mgO_2_/L were 1.80 and 1.77 times (S.I.), and 2.79 and 2.23 times (S.IV.) lower, respectively. When the tests were conducted at the lowest of the analysed DO concentrations (1.0 mgO_2_/L), at each of those stages the value of the analysed indicator was higher than when the test was conducted for 1.5 mgO_2_/L. In series II, the value of AOR-SB_V.I.1.5._ was 1.18 times higher than that obtained in tests for variant II. The observations suggest that ammonia-oxidizing microorganisms could develop in activated sludge flocs showing higher activity in the conditions of lower DO concentration (Park et al. 2002). According to the study by Park et al. (2002), such microorganisms include *Nitrosospira* and *Nitrosomonas oligotropha* lineages. Lower DO concentration is also one of the factors considered favourable for the recently investigated Comammox bacteria (Roots et al. 2019).

### Effect of the pre-set conditions of operation of IFAS-MBSBBR on the ammonia oxidation rate and nitrite oxidation rate

Conducting batch tests with the assumption that oxygen concentration would be approximate to saturation at 20 °C aimed at limiting the effect of DO concentration on the ammonia and nitrite oxidation rate. The tests were conducted at the end of each series designated in the experiment, allowing for a comparison of the effect of all changes introduced in subsequent series.

After reducing the duration of aerated sub-phases from 30 min (S.I.) to 20 min (S.II.), a 1.77 times decrease was recorded in the value of AOR-SB_V.II._ (from 6.841 mgN–NH_4_^+^/gVSS·h to 3.856 mgN–NH_4_^+^/gVSS·h) and a 2.07 times increase in the values of the analysed indicator in the case of biofilm (from 3.374 mgN–NH_4_^+^/gVSS·h to 6.993 mgN–NH_4_^+^/gVSS·h). The activity of ammonia-oxidizing bacteria in activated sludge therefore decreased, with a simultaneous increase in their activity in biofilm. Moreover, the value recorded in series I for activated sludge was approximate to that evidenced for biofilm in series II, and the value of AOR-B_V.II._ in series I corresponded with that recorded for activated sludge after the change in the aeration strategy. In the case of NitOR, a decrease in the analysed value for activated sludge was also observed, and its increase in tests conducted for biofilm. The NitOR-SB decreased 1.20 times (from 4.211 mgN–NO_2_^−^/gVSS·h to 3.503 mgN–NO_2_^−^/gVSS·h), whereas NitOR-B increased 2.32 times (from 9.239 mgN–NO_2_^−^/gVSS·h to 21.446 mgN–NO_2_^−^/gVSS·h). In series II, the nitrite nitrogen oxidation rate in the biofilm was the highest throughout the experiment, possibly due to the largest relative abundance of *Nitrospira* in biofilm recorded in the microbiological analysis (Godzieba et al. 2022).

Such observations lead to the conclusion that ammonia- and nitrite-oxidizing microorganisms inhabiting each of the discussed forms of biomass showed different responses to the introduced changes in the reactor’s aeration strategy. Interestingly, although the reduction in the duration of aerated sub-phases resulted in the occurrence of more sub-phases with the air pumps switched off in aerated phases (S.I.–8 vs. S.II.–11), the NitOR in the case of biomass in the form of biofilm increased. Considering study results by Yang and Yang (2011) who conducted research in a moving bed membrane bioreactor with simultaneous nitrification and denitrification, an increase in the R value was expected to cause suppression of NOB bacteria, observed as a decrease in the value of NitOR.

A reduction in the reactor’s organic and nitrogen loading rate introduced in series III caused a 1.33-fold increase in AOR for activated sludge, whereas in tests conducted for biofilm, a 1.49-fold decrease was recorded. The observations point to an increase in the activity of ammonia-oxidizing bacteria that developed in activated sludge flocs, with a simultaneous decrease in the activity of such bacteria inhabiting biofilm. In series III, results of NitUR tests also showed a decrease in NitOR-B, suggesting a decrease in the activity of NOB bacteria developing in the form of biofilm. Presumably, the ammonia nitrogen supplied with raw wastewater to IFAS-MBSBBR was first oxidized by the microorganisms inhabiting activated sludge, resulting in insufficient load supplied to the microorganisms inhabiting biofilm, causing a decrease in their population. It could also be presumed that oxygen and substrate diffusion within biofilm was of considerable importance here. Microbiological analysis also showed that the relative abundance of *Nitrospira* in the biofilm then decreased (Godzieba et al. 2022).

Due to the return to the values of L_COD_ and L_N_ assumed at the beginning of the experiment in reference to the values recorded in series III, for activated sludge, an increase in AOR (1.58-fold) and NitOR (1.26-fold) was observed and in the case of biofilm a decrease in both of the analysed values 1.30 times (AOR-B) and 1.04 times (NitOR-B), respectively. The predefined reactor’s operation conditions therefore contributed to an increase in the activity of nitrification bacteria in activated sludge and its decrease in biofilm.

### Effect of free ammonia and free nitrous acid on changes in the activity of particular groups of nitrifying microorganisms

The concentration of free ammonia (FA) and free nitrous acid (FNA) may affect the kinetics of the nitrification process. According to Jiang et al. (2019), nitrite-oxidizing bacteria are more sensitive to FA than AOB. It is reported that NOB can be inhibited by FA at about 0.1 to 1 mgFA/L, whereas AOB inhibition threshold was 10–150 mgFA/L. Some previous works reported that nitritation was achieved even at 20 mgFA/L (Chung et al. 2007).

In the AUR tests, the FA concentration was within a range of 0.349–0.474 mgFA/L, significantly lower than the thresholds reported for the inhibition of AOB (Table 6). These values, however, may have had an effect on bacteria capable of oxidizing nitrite nitrogen. During the test conducted for activated sludge, accumulation of nitrite nitrogen was observed which can be equated with the occurrence of NOB suppression. The ratio between nitrite increase and ammonia loss (RNIAL) in each of the tests conducted for this form of biomass exceeded 21.98% (except S.II. when the test was conducted at unlimited DO). The highest accumulation of N–NO_2_^−^ was observed in AUR-SB in series S.I. when oxygen concentration was maintained at a level of 1.5 mgO_2_/L. Almost 50% of the initial N–NH_4_^+^ was accumulated as nitrite. Concentration of FA was then 0.375 mgFA/L. In the case of the tests conducted for the biofilm, none of them showed significant accumulation of N–NO_2_^−^ (RNIAL < 1%), although the FA concentration was slightly higher than in tests carried out for other forms of biomass. In S.I., certain nitrite accumulation was also observed in the test for hybrid. As mentioned before, in the same series, the highest N–NO_2_^−^ accumulation in AUR-SB was recorded. This may give rise to the assumption that in the case of simultaneous tests carried out for activated sludge and biofilm, NOB suppression in activated sludge flocs resulted in the accumulation of nitrite. The observations made in this study suggest that the observed partial inhibition of NOB could be influenced by the concentration of FA at a level of 0.349 mgFA/L.

**Table 6:** Free ammonia (FA) and free nitrous acid (FNA) at the beginning of batch tests

Parameter	Type of biomass	Series I			Series II		Series III	Series IV		
		DO [mgO_2_/L]			DO [mgO_2_/L]		DO [mgO_2_/L]	DO [mgO_2_/L]		
		nl	1.5	1.0	nl	1.5	nl	nl	1.5	1.0
AUR test Initial concentration of substrate [mgN–NH_4_^+^/L]	**SB**	15.90	15.90	15.00	15.20	16.10	14.80	18.00	15.30	15.80
	**B**	16.50	18.70	18.70	18.20	20.10	17.30	17.30	18.30	18.60
	**H**	–	17.60	–	–	19.90	–	–	17.40	–
RNIAL [%]	**SB**	26.54	49.83	30.31	10.17	21.98	24.08	32.25	31.63	29.28
	**B**	1.03	0.36	0.47	0.18	0.19	0.17	0.31	0.92	0.25
	**H**	–	12.80	–	–	3.85	–	–	3.82	–
NitUR test Initial concentration of substrate [mgN–NO_2_^−^/L]	**SB**	17.80	18.10	–	14.80	16.10	14.80	15.60	15.40	–
	**B**	17.90	20.60	–	16.90	18.30	18.00	15.10	17.60	–
	**H**	–	20.00	–	–	18.90	–	–	17.40	–
FA [mg/L] (AUR test)	**SB**	0.375	0.375	0.354	0.358	0.380	0.349	0.424	0.361	0.373
	**B**	0.389	0.441	0.441	0.429	0.474	0.408	0.408	0.431	0.439
	**H**	–	0.415	–	–	0.469	–	–	0.410	–
FNA [mg/L] (NitUR test)	**SB**	2.994·10^–3^	3.044·10^–3^	–	2.489·10^–3^	2.708·10^–3^	2.489·10^–3^	2.624·10^–3^	2.590·10^–3^	–
	B	3.010·10^–3^	3.464·10^–3^	–	2.842·10^–3^	3.078·10^–3^	3.027·10^–3^	2.539·10^–3^	2.960·10^–3^	–
	H	–	3.364·10^–3^	–	–	3.179·10^–3^	–	–	2.926·10^–3^	–

FNA is considered a universal NOB inhibitor, but its concentration determines to what extent it will affect individual types of nitrifying bacteria differing in terms of resistance (Meng et al. 2022). According to Pedrouso et al. (2017), *Nitrospira* (belonging to NOB) completely inhibits FNA concentration at a level of 0.02 mgFNA/L, while in the case of *Nitrobacter* (belonging to AOB), it is 20 times higher, i.e. 1 TN/L (Blackburne et al. 2007). FNA concentrations in the conducted tests remained significantly below the threshold values reported in the literature.

### Energy use

Most of the energy supplied to a wastewater treatment plant is used for aeration, necessary for biochemical oxidation of organic compounds and nitrite oxidation. According to Luo et al. (2019), in conventional wastewater treatment plants, from 25% to 60% of operational costs are associated with energy use, whereas aeration accounts for 50–75% of total energy expenditure of wastewater treatment plants.

The methodology proposed by the cited authors (Luo et al. 2019) provided the basis for the calculation of energy consumption (Eq. 5)—E_O_ (kWh/kg):

5 $${\text{E}}_{{\text{O}}} = \frac{{{\text{E}}_{{\text{d}}} }}{{\Delta {\text{m}}}}{ }\left[ {{\text{kWh}}/{\text{kg}}} \right]$$

E_d_ is daily electric energy consumption (kWh/d), and Δm [kg/d] was calculated as a product of Q (volume of wastewater flow (m^3^/d)), $\Delta {\text{C}}_{{{\text{COD}}}}$, $\Delta {\text{C}}_{{{\text{N}} - {\text{NH}}_{4}^{ + } }}$ (differences in COD and N–NH_4_^+^ concentrations in influent and effluent with consideration of N–NH_4_^+^ released in the ammonification process (g/m^3^)), and 4.18 (oxygen consumption for 1 g of N–NH_4_^+^ oxidation) (Eq. 6):

6 $$\Delta {\text{m}} = \frac{{{\text{Q}} \cdot \Delta {\text{C}}_{{{\text{COD}}}} + 4.18 \cdot {\text{Q}} \cdot \Delta {\text{C}}_{{{\text{N}} - {\text{NH}}_{4}^{ + } }} }}{1000}{ }\left[ {{\text{kg}}/{\text{d}}} \right]$$

**Fig. 2:**
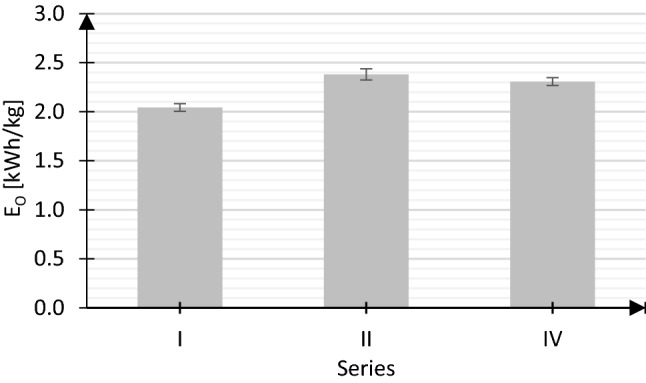
Average energy consumption (E_O_)

The comparison of E_O_ values covered series in which IFAS-MBSBBR operated with identical reactor’s organic and nitrite loading rate (Fig. 2). Due to the reduction in the duration of aerated sub-phases from 30 min (S.I.) to 20 min (S.II., S.IV.), the total duration of aerated sub-phases in a day decreased from 780 min to 690 min. Contrary to expectations, however, the duration of the operation of air pumps in a day increased by 16% (S.II. vs. S.I.) and 13% (S.IV. vs. S.I.). The consequence was higher Eo value in series II and IV in comparison with that determined in series I. In the analysed system, an increase in the value of R therefore determined the amount of energy used for aeration. Different conclusions were drawn by Singh et al. (2018), conducting research in a field-scale IFAS reactor. With an increase in the R value, they observed a decrease in energy used for powering the air pumps. In the analysed system, however, there was no established DO set-point. Due to that, in aerated sub-phases, air pumps operated continuously throughout the sub-phases, and a reduction in the duration of aerated sub-phases in subsequent series was directly related to the reduction in the time of operation of air pumps. The obtained results showed that in the case of systems with intermittent aeration in which the operation of air pumps is controlled through switching them on and off not only according to the adopted R value, but also the assumed oxygen concentration (it is worth emphasizing that such a control system is usually applied in wastewater treatment plants), it cannot be assumed that a reduction in the duration of aerated sub-phases will permit a decrease in energy use for aeration.

Considering the average price of 1 kWh of energy in Warsaw (Poland)—0.79 PLN (0.17 €), the costs of aeration for removal of pollutants increased from 1.61 PLN/kg (0.35 €/kg) (S.I.) to 1.88 PLN/kg (0.41 €/kg) (S.II.), 1.82 PLN/kg (0.40 €/kg) (S.IV.). At the scale of 1 year, it would generate approximately 14–15% higher costs related to energy used for powering air pumps.

### N_2_O emission

Due to the high value of the global warming potential (GWP), nitrous oxide is considered as one of significant greenhouse gases. Its value is almost 300 times higher than that determined for carbon dioxide (Schreiber et al. 2012). Papers focusing on N_2_O emission in wastewater treatment plants operating in the moving bed hybrid technology, however, are scarce (Lo et al., 2010; Sabba et al. 2018;). The processes of biological nitrogen removal (BNR) through nitrification and denitrification (N/D) are considered the primary sources of N_2_O emission, due to the fact that both autotrophic and heterotrophic bacteria can be responsible for the production of nitrous oxide. In the nitrification process, nitrous oxide is produced as a by-product of ammonia oxidation by AOB bacteria in two different ways: 1) incomplete oxidation of hydroxylamine (NH_2_OH) to nitrite and 2) reduction in nitrite as an acceptor of electrons to N_2_O (autotrophic denitrification conducted by AOB bacteria). N_2_O can also be a by-product of incomplete heterotrophic denitrification (Liu et al. 2021).

According to Su et al. (2017) and Zheng et al. (2021), the parameters of operation of a wastewater treatment plant such as low DO concentration and the aeration strategy have a considerable effect on the N_2_O emission. An effective way to reduce N_2_O emission proved to be the introduction of IA. According to Ni et al. (2013), increasing the cycle frequency of periodic aeration may cause a reduction in N_2_O emission.

Liu et al. (2021), investigating N_2_O emission in two activated sludge SBR reactors, observed lower N_2_O emission for the system operating with IA SBR1 (average 0.19%) than for that operating with continuous aeration (SBR2) (average 0.42%) (Table 7). The analysis of study results of Lo et al. (2010) suggests that the amount of N_2_O emission also depends on the form of biomass in the reactor and DO concentration. In their research in a hybrid SBR reactor for simultaneous nitrification, denitrification, and phosphorus removal, the cited authors conducted an experiment for the hybrid system, then separated activated sludge and biofilm to different reactors, and analysed the emission of N_2_O in each of these solutions. The highest N_2_O emission was recorded when the reactor operated as a hybrid—21.2%. When particular forms of biomass were separated, N_2_O emission reached 0.5% for biofilm and 4.2% for activated sludge, respectively. Low emission of N_2_O in tests conducted for biofilm was associated with the fact that a higher abundance of microorganisms inhabiting this form of biomass was constituted by heterotrophic bacteria and the internal part of the biofilm provided for anoxic conditions suitable for N_2_O reduction (Table 7).

**Table 7:** N_2_O emission factors in various systems with nitrification/denitrification

Type of reactor and configuration	Biomass form	N_2_O emission factor (%)	References
Fully aerobic SND sequencing batch air-lift reactor	Aerobic granule	21.9 ± 7.1	Zhang et al. (2015)
Anoxic-aerobic SND sequencing batch air-lift reactor (R = 1/5; 225 min:45 min)	Aerobic granule	7.0 ± 1.6	
Step-feeding multiple A/O SBR (R = 1; 30 min:30 min)	Activated sludge	4.38	Sun et al. (2018)
One-feeding multiple A/O SBR (R = 1; 30 min:30 min)	Activated sludge	4.66	
multiple anoxic-aerobic SBR—600 mL/min (R = 1; 30 min:30 min)	Activated sludge	10.1	Wang et al. (2016)
multiple anoxic-aerobic SBR—1200 mL/min (R = 1; 30 min:30 min)	Activated sludge	2.3	
Intermittently aerated SBR1 (R = 1; 30 min:30 min)	Activated sludge	0.01–0.53	Liu et al. (2021)
Continuously aerated SBR2	Activated sludge	0.11–2.09	
Continuously aerated Hybrid SBRs	Biofilm	0.4	Lo et al. (2010)
	Activated sludge	4.2	
	Hybrid	21.2	
Intermittently aerated IFAS-MBSBBR R = 1/3 (30 min:10 min)	Hybrid	0.896	This study
Intermittently aerated IFAS-MBSBBR R = 1/2 (20 min:10 min)	Hybrid	1.091	

In this study, reducing the duration of aerated sub-phases from 30 min (S.I.) to 20 min (S.II.) caused an increase in N_2_O emission from 0.896% to 1.091%. The obtained values are, respectively, 4.72 times and 5.74 times higher than those determined for activated sludge with intermittent aeration by Liu et al. (2021). This suggests that the form of biomass in the reactor has a considerable effect on the analysed phenomenon. Differences between the values recorded in this study and those determined for the hybrid system by Lo et al. (2010) are almost 24 (S.I.) and 20 (S.II.) times lower. It could have been determined by oxygen concentration values and the applied aeration strategy, because they conducted the experiment with continuous aeration and DO reaching 0–1.5 mgO_2_/L. Microbiological analysis done by Godzieba et al. (2022) showed that bacteria able to conduct autotrophic denitrification, one of potential sources of N_2_O, also developed in the system. The biomass contained e.g. *Hydrogenophaga sp., Paracoccus*, *Thauera,* and *Rhodobacter*, but there was no increase in any of them between the two analysed series.

According to Polish legislation, the fee for the emission of 1 mgN_2_O in 2022 is 94.36 PLN (20.56 €). With a reduction in the duration of non-aerated sub-phases from 30 min to 20 min, the average payment for the emission of one of the primary greenhouse gases from the discussed system in 1 day would increase by almost 32% from 51.53 PLN/d (11.23 €/d) to 67.60 PLN/d (14.73 €/d).

To sum up, this study evidenced that in the IFAS-MBSBBR system with intermittent aeration at R = 1/3 and DO = 1.5 mgO_2_/L, next to a reduction in costs related to aeration, lower N_2_O emission is also obtained in comparison with the system operating at the same DO and R = 1/2.

## Conclusions

- The greatest ammonia oxidation activity was evidenced in activated sludge flocs, while the activity of nitrite nitrogen oxidation was higher for biofilm.
- The response of ammonia-oxidizing bacteria to the reduction in the reactor’s organic and nitrogen loading rate was stronger than that of nitrite-oxidizing bacteria.
- A reduction in the duration of aerated sub-phases in a system with intermittent aeration and air pumps controlled through switching them on and off according to both the adopted R value and the assumed oxygen concentration caused no decrease in energy use for aeration. Lower energy use was recorded when the duration of aerated sub-phases was longer.
- In intermittently aerated IFAS-MBSBBR, lower N_2_O emission was recorded when the reactor operated with a longer duration of aerated sub-phases.

## Supplementary Information

Below is the link to the electronic supplementary material.

Supplementary file1 (DOCX 138 KB)
