# Supplementary material for: Nitrification kinetics, N2O emission, and energy use in intermittently aerated hybrid reactor under different organic loading rates
Source: Int J Environ Sci Technol (Tehran). 2022 Dec 20:1–14. Online ahead of print. doi: 10.1007/s13762-022-04715-6 (PMC9765392; doi:10.1007/s13762-022-04715-6)
Supplement: Supplementary file 1 — Supplementary file1 (DOCX 138 KB) [file 13762_2022_4715_MOESM1_ESM.docx]

**Supplementary material**

Nitrification kinetics, N_2_O emission and energy use in intermittently aerated hybrid reactor under different organic loading rates.

**International Journal of Environmental Science and Technology**

Olga Zajac^a,*^ and Monika Zubrowska-Sudol^a^

^a^ Warsaw University of Technology, Faculty of Building Services, Hydro and Environmental Engineering, Department of Water Supply and Wastewater Treatment, Nowowiejska 20, 00 653 Warsaw, Poland

* Corresponding author. E-mail address: [olga.zajac.dokt@.pw.edu.pl](mailto:olga.zajac.dokt@.pw.edu.pl) (O. Zajac)

**Supplementary material** - **- Influent and effluent characteristics of IFAS-MBSBBR**

**Figure 1**. Influent and effluent concentrations and removal efficiencies of ammonia nitrogen

**Figure 2**. Effluent concentrations of nitrite and nitrate nitrogen

**Figure 3**. Influent and effluent concentrations and removal efficiencies of total nitrogen

**Figure 4**. Influent and effluent concentrations and removal efficiencies of COD

**Figure 5**. Influent and effluent concentrations and removal efficiencies of phosphorus

**Supplementary material** - **- Results of particular AUR and NitUR tests**

a)

b)

**Figure 6.** N-NH_4_^+^, N-NO_2_^-^, N-NO_3_^-^ profiles during test (a) AUR-SB (b) NitUR-SB. Series I

a)

b)

**Figure 7.** N-NH_4_^+^, N-NO_2_^-^, N-NO_3_^-^ profiles during test (a) AUR-B (b) NitUR-B. Series I

a)

b)

**Figure 8.** N-NH_4_^+^, N-NO_2_^-^, N-NO_3_^-^ profiles during test (a) AUR-SB (b) NitUR-SB. Series II

a)

b)

**Figure 9.** N-NH_4_^+^, N-NO_2_^-^, N-NO_3_^-^ profiles during test (a) AUR-B (b) NitUR-B. Series II

a)

b)

**Figure 10.** N-NH_4_^+^, N-NO_2_^-^, N-NO_3_^-^ profiles during test (a) AUR-SB (b) NitUR-SB. Series III

a)

b)

**Figure 11.** N-NH_4_^+^, N-NO_2_^-^, N-NO_3_^-^ profiles during test (a) AUR-B (b) NitUR-B. Series III

a)

b)

**Figure 12.** N-NH_4_^+^, N-NO_2_^-^, N-NO_3_^-^ profiles during test (a) AUR-SB (b) NitUR-SB. Series IV

a)

b)

**Figure 13.** N-NH_4_^+^, N-NO_2_^-^, N-NO_3_^-^ profiles during test (a) AUR-B (b) NitUR-B. Series IV
